# Electrocardiographic P-wave Indices as a Useful Tool to Predict Successful Percutaneous Balloon Mitral Valvotomy in Patients with Mitral Stenosis

**DOI:** 10.5681/jcvtr.2014.002

**Published:** 2014-03-21

**Authors:** Babak Kazemi, Ali Rostami, Naser Aslanabadi, Samad Ghaffari

**Affiliations:** Cardiovascular Research Center, Tabriz University of Medical Sciences, Tabriz, Iran

**Keywords:** Mitral Stenosis, P-wave Duration, P-wave Dispersion

## Abstract

***Introduction***: Patients with hemodynamically significant mitral stenosis (MS) have prolonged P-wave duration and increased P-wave dispersion (PWD) that decrease after successful percutaneous balloon mitral valvotomy (PBMV). The purpose of this study was to investigate if the changes in these indices may predict a successful procedure.

***Methods***: Fifty two patients with MS in sinus rhythm underwent PBMV (90.4% female; mean age 38±10 years). Mitral valve area (MVA), valve score, mean diastolic mitral gradient (mMVG), mitral regurgitation severity, and systolic pulmonary artery pressure (sPAP) were evaluated by echocardiography before PBMV and repeated after one month. P-wave duration (P_max_ /P_min_) and PWD were measured before and immediately after PBMV, at discharge, and at the end of the first month after discharge.

***Results***: Among all procedures, 38 (73.1%) were defined as successful. Mean age, valve score, mMVG, and MVA before PBMV were similar for both groups. MVA was significantly greater in the successful PBMV group (1.65±0.27 vs. 1.41±0.22; P= 0.003). sPAP was reduced after PBMV in all patients and there were no significant differences in the mean sPAP before and after PBMV in both successful and unsuccessful groups. P_max_ and PWD were significantly decreased immediately after the procedure (P= 0.035), the next day (P= 0.005) and at one month (P= 0.002) only in patients with successful PBMV. P_min_ did not change significantly in either group.

***Conclusion***: Only is successful PBMV associated with a decrease in P_max_ and PWD. These simple electrocardiographic indices may predict the success of the procedure immediately after PBMV.

## Introduction

P-wave duration (P_max _/P_min_) and P-wave dispersion (PWD) are electrocardiographic (ECG) indices that recently have received increasing attention and been examined in a broad range of clinical settings. The prolongation of intra- and inter-atrial conduction time, and inhomogeneous propagation of sinus impulses are well-known electrophysiologic characteristics of the atrium prone to fibrillate.^1,2^ PWD has been associated with these characteristic changes in the atria and used for noninvasive surrogate detection of atrial electrophysiology.^1-4^ It can be defined as the difference between P_max _and P_min_.

Rheumatic mitral stenosis (MS) is frequently seen in developing countries and causes significant morbidity and mortality.^5^ Atrial fibrillation is the most common sustained arrhythmia encountered in patients with rheumatic MS. Limited studies are available in the literature on the relation between MS and P-wave indices. First, Turhan et al.^6^ evaluated the effect of PBMV on PWD in 29 patients with MS and concluded that PWD is significantly higher in patients with MS than in healthy control subjects, and it decreases significantly after PBMV both in the short and long term. Then, Guntekin et al.^7^ followed 30 patients with mild to moderate MS with ECG and echocardiography and showed that P_max_ and PWD increase progressively in accordance with the severity of MS.

The purpose of this study was to investigate for the first time if the immediate changes in these P-wave indices could confirm a successful PBMV procedure and differentiate it from an unsuccessful one in patients with hemodynamically significant MS.

## Materials and methods

### Patient selection

From October 2012 to October 2013, 153 patients with moderate to severe MS were admitted for PBMV to Madani Heart Center, Tabriz, Iran. Sixty five (42.4%) patients who were in sinus rhythm were initially recruited in this study. Patients who had coronary artery disease (n= 0), hypertension (n= 2), diabetes mellitus (n= 2), hyperthyroidism (n= 0), pericardial effusion (n= 0), chronic obstructive pulmonary disease (n= 0), ventricular preexcitation (n= 0), bundle branch block (n= 0), atrioventricular conduction abnormalities (n= 0), abnormal serum electrolytes (n= 0), history of documented AF (n= 0), developed AF during the study (n= 2), needed valvular intervention during follow-up (n= 1), or patients lost to follow-up (n= 6) were excluded from the study. None of them were taking type I or type III antiarrhythmic agents. Beta-blockers, digitalis, aldosterone antagonists, angiotensin receptor antagonists, and diuretics which can affect atrial structural and electrophysiologic remodeling were withheld for at least 5 half-times before the procedure.

### Echocardiographic evaluation

The echocardiographic examination was performed at rest, with the patient at left lateral decubitus position, using a commercially available echocardiographic device (Vivid 7, General Electric, Milwaukee, WI, USA) with a 3-MHz transducer, by two experienced cardiologists who were blinded to the clinical data. Transesophageal and transthoracic echocardiography were performed less than 24 hours before the procedure. Transthoracic echocardiography was repeated one month after PBMV. Mitral valve anatomy was scored on the basis of the Wilkins echo scoring system.^8^ Mitral valve area was calculated by planimetry or, in the absence of significant mitral regurgitation (MR), from pressure half-time.^9^ Semiquantitative estimation of MR (mild, moderate, or severe) was made with color flow mapping in parasternal long axis and apical 4-chamber views. The mean transmitral diastolic mitral valve gradients (mMVG) were also calculated with Doppler ultrasound scanning studies. Systolic pulmonary artery pressure (sPAP) was calculated using the Bernoulli equation from tricuspid insufficiency flow in the parasternal short axis and apical 4-chamber views, and the highest tricuspid regurgitation velocity was taken as the study sample. A sPAP< 30 mmHg was considered normal. Pulmonary arterial hypertension (PAH) was defined as mild (sPAP= 30-44 mmHg), moderate (sPAP= 45-59 mmHg), and severe (sPAP≥ 60 mmHg). All values were measured on three separate beats and then averaged for all parameters. Intra- and inter-observer coefficients of variation for echocardiographic parameters were found to be less than 5% and nonsignificant. We defined the PBMV successful at the one month follow-up transthoracic echocardiography if a valve area increment ≥50% or a final valve area >1.5 cm^2^, with no more than moderate MR is achieved. We divided the patients into two groups according to this definition: successful and unsuccessful PBMV.

### PBMV

Our technique for this procedure has been described previously.^10^ Briefly, it was performed in a fasting state under local anesthesia and mild sedation by the antegrade trans-septal approach with a stepwise dilation technique using Inoue balloon catheter (Toray Industries, Inc., Tokyo, Japan), beginning with a small diameter and re-evaluating the transmitral gradient and degree of MR after each inflation. Right and left heart pressure measurements, including simultaneous left atrial (LA) and left ventricular pressures, were obtained before and after PBMV. The procedure was terminated once a satisfactory hemodynamic result was achieved, defined as a decrease of at least one-half of the initial transmitral gradient with no further reduction despite 2 more dilation using balloon sizes in 0.5–1-mm increments.

### ECG measurements

The GE MAC 500 System (Soma Technology, Inc., USA) was used for ECG acquisition. ECG was recorded for each patient 1 day before PBMV and repeated immediately after PBMV, before discharge, and at the end of the first month after discharge. The ECGs were numbered and presented to the analyzing investigators without name and date information. All measurements of P_max _/P_min_ were made blindly by 2 experienced investigators and were measured manually in all simultaneously recorded 12 leads of the surface ECG which was taken at a rate of 50 mm/sec with 1 mV/cm standardization. The mean P_max _/P_min_ for at least 3 complexes were calculated in each lead and their average values were used for groups comparisons. For greater accuracy, measurements were performed with calipers and magnifying lens, as described by previous investigators.^2,11^ The onset of the P wave was defined as the point of first visible upward departure from baseline for positive waveforms, and as the point of first downward departure from baseline for negative waveforms. The return to the baseline was considered to be the end of the P wave. The P_max_ measured in any of the 12 leads of the surface ECG was used as the longest atrial conduction time. The difference between the P_max_ and the P_min_ was calculated and defined as PWD.^2^ Intra- and inter-observer coefficients of variation for P wave indices were found to be less than 5% and nonsignificant.

### Statistical Analysis

Data were expressed as the mean ± SD. Groups were compared by the Student’s t-test for the continuous variables, the χ^2^ test for qualitative variables, and with the Mann-Whitney U-test for the variables without normal distribution. Baseline and follow-up echocardiographic and ECG parameters were compared by paired t-tests. Pearson correlation coefficient analysis was used to assess the relationship among variables. A two-tailed P value<0.05 was considered significant. All statistical analyses were conducted with SPSS 17 (SPSS Inc., Chicago, IL).

## Results

Ultimately, 52 patients (5 men, 47 women; mean age 38±10 years; range: 20 to 65 years) were enrolled in this study. Among these patients, 38 (73.1%) experienced a successful PBMV according to our definition. The reasons for unsuccessful PBMV were the occurrence of MR> 2^+^ in 5 patients, and MR> 2^+^ with MVA< 1.5 cm^2^ in 4 patients and less than 50% increase in MVA in 5 patients. The mean Wilkins echo score in all patients was 8.5±1.2 (range: 5-12). Since the mean valvular score of our patients were relatively high we reanalyzed them according to a score≤ 8 (16 patients) or >8 (34 patients). PBMV was successful in 78.9% of those with a score≤ 8 with respect to 67.6% in those with a score> 8.

The MVA was 0.90±0.2 cm^2^ (range: 0.5-1.4 cm^2^) which increased to 1.59±0.2 cm^2^ (range: 1.1-2.3 cm^2^) after PBMV (Table 1). Mean age (37.7±10.8 vs. 40.2±10 years; *P*= 0.44), mitral valve score (8.4±1.37 vs. 8.78±0.72; *P*= 0.20), mMVG (12.01±4.5 vs. 12.03±3.26 mmHg; *P*= 0.98), and MVA (0.87±0.23 vs. 0.97±0.14 cm^2^; *P*= 0.60) before PBMV were similar for successful and unsuccessful procedures. There was a trend for greater reduction in mMVG after successful PBMV but it did not reach statistical significance (6.5±2.4 vs. 8.05±2.8 mmHg; *P*= 0.09). MVA was significantly greater in the successful PBMV group (1.65±0.27 vs. 1.41±0.22 cm^2^; *P*= 0.003) (Table 1).

**Table 1:** Demographic and echocardiographic finding before and after PBMV

**Variables**	**All patients (n= 52)**	**Successful PBMV** **(n= 38)**	**Unsuccessful PBMV** **(n= 14)**	*P*
Age (years)	38.4±10.5	37.7±10.8	40.2±10	0.44
Valvular score	8.5±1.2	8.4±1.37	8.78±0.72	0.20
Pre-PBMV^*^ MVA^†^ (cm^2^)	0.90±0.2	0.87±0.23	0.97±0.14	0.60
Post-PBMV MVA (cm^2^)	1.59±0.2	1.65±0.27	1.41±0.22	0.003
Pre-PBMV MVG^‡^ (mmHg)	12.0±4.1	12.01±4.5	12.03±3.26	0.98
Post-PBMV MVG (mmHg)	6.9±2.6	6.5±2.4	8.05±2.8	0.09
Pre-PBMV PAP^§^ (mmHg)	46.8±14.1	46.5±15.3	47.5±10.3	0.78
Post-PBMV PAP (mmHg)	30.7±11.5	29.3±11.8	34.4±10.2	0.14

*percutaneous balloon mitral valvotomy; ^†^mitral valve area; ^‡^mitral valve gradient; ^§^pulmonary artery pressure.

**Figure 1:**
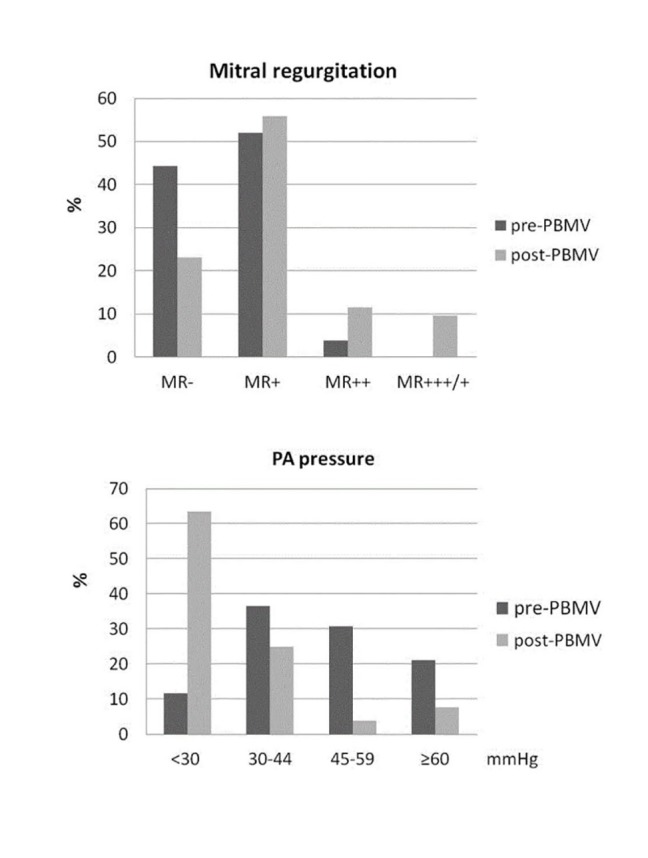
Frequency of MR and PAH in all patients before and after PBMV (see text for details).

MR was present in 55.8% of patients (mild and moderate in 51.9% and 3.9%, respectively) before PBMV. At one month follow-up 77% of patient had MR (mild, moderate, and severe in 55.7%, 11.5%, and 9.6%, respectively) (Figure 1).

**Figure 2:**
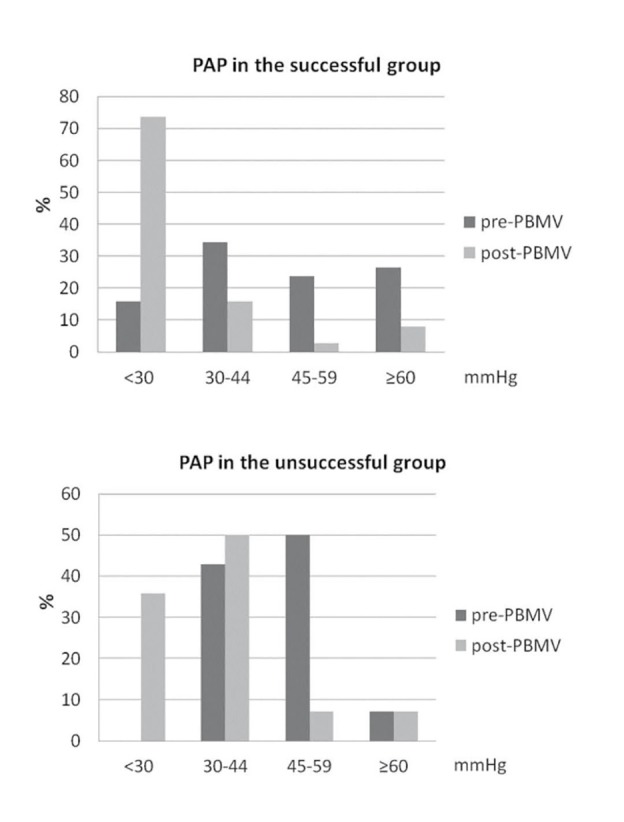
Frequency of PAH in patients before and after successful and unsuccessful PBMV (see text for details).

PAH was present in 88.5% of patients before PBMV: mild, moderate, and severe PAH were present in 36.5%, 30.7%, and 21.1% of patients, respectively (Figure 1). sPAP was reduced after PBMV in all patients and there was no significant difference in the mean of sPAP before and after PBMV in both successful and unsuccessful groups (Table 1). At one month after discharge 63.5% of patients had a normal sPAP. Mild, moderate, and severe PAH were present in 25%, 3.8%, and 7.7%, respectively. In those who had a successful PBMV, 15.8% had a normal sPAP and mild, moderate, and severe PAH were present in 34.2%, 23.7%, and 26.3% of patients, respectively before the procedure. After PBMV, 73.7% of patients in this group had a normal sPAP and mild, moderate, and severe PAH were present in 15.8%, 2.6%, and 7.9% of patients, respectively (Figure 2). In patients who experienced an unsuccessful PBMV, no one had a normal sPAP and mild, moderate, and severe PAH were present in 42.9%, 50%, and 7.1% of patients, respectively before the procedure. After PBMV, 35.7% of patients in this group had a normal sPAP and mild, moderate, and severe PAH were present in 50%, 7.1%, and 7.1% of patients, respectively (Figure 2).

P_max _and PWD were significantly decreased immediately after the procedure (*P*= 0.035 and 0.02, respectively), the next day (*P*= 0.005 and 0.002, respectively) and at one month (*P*= 0.002 and 0.001, respectively) only in patients with successful PBMV (Table 2). P_min_ did not change significantly in the successful group after PBMV and during follow-up. Patients with unsuccessful PBMV did not show any significant change in any P-wave indices after PBMV and one month follow-up. In fact PWD was increased at one month but it did not reach statistical significance (Table 3). In patients with a successful PBMV the decreases in P_max _and PWD were not significantly correlated with improvements of echocardiographic variables [MVA (r=0.005, *P*=0.97) and mMVG (r=-0.49, *P*=0.76)] at the end of the first month follow-up.

**Table 2:** P-wave indices before and after successful PBMV

**Indices (msec)**	**Before PBMV** ^†^ **mean±SD **	**Immediately after ** ** mean±SD ** **(** *P* **)**	**Before discharge** **mean±SD ** **(** *P* **)**	**At one month** **mean±SD** **** **(** *P* **)**
P_max _	116±13	112±9 (0.035)*	110±11 (0.005)*	109±10 (0.002)*
P_min _	77±10	77±8.2 (0.72)	75±8 (0.39)	76±10 (0.74)
PWD	38.6±12	35.1±10.9 (0.02)*	34.3±11 (0.002)*	32.7±11.5 (0.001)*

*p-value is significant; ^†^percutaneous balloon mitral valvotomy.

**Table 3:** P-wave indices before and after unsuccessful PBMV

**Indices (msec)**	**Before PBMV** ^*^ **mean±SD **	**Immediately after** **mean±SD ** **(** *P* **)**	**Before discharge** **mean±SD ** **(** *P* **)**	**At one month** **mean±SD ** **(** *P* **)**
P_max _	120±18	118±16 (0.59)	117±20 (0.42)	117±8 (0.56)
P_min _	78±13	77±10 (0.64)	79±13 (0.91)	73±11 (0.17)
PWD	40.7±12.9	41±14 (0.90)	38.2±12 (0.43)	44.2±10.7 (0.22)

*percutaneous balloon mitral valvotomy

## Discussion

The major finding of this study was that in patients with moderate to severe MS only successful PBMV was associated with a decrease in P_max_ and PWD. These simple ECG indices may predict the success of the procedure immediately after PBMV.

Dilaveris at al.^2^ was the first to describe PWD as the difference between maximum and minimum P-wave duration measured on the standard 12-lead surface ECG and showed that it is a marker of inhomogeneous and discontinuous propagation of sinus impulses.^1,2^ They also observed that increased PWD carries an increased risk for AF.^2^ Furthermore, the correlation between inter-atrial and intra-atrial conduction abnormalities and the induction of AF has been well documented.^12,13^

Magnany et al.^14^ have excellently reviewed the vast clinical conditions studied utilizing P-wave indices. These included about 40 studies from cardiovascular diseases and risk factors, noninvasive measures, non-cardiac conditions, and especially AF. However, there are only 5 studies ^6,7,15-18^ on the relation between MS and atrial conduction /refractoriness in the literature. Soylu et al.^16^ studied hemodynamic and electrophysiologic changes in 25 patients undergoing PBMV. They showed that relief of chronic atrial stretch results in an immediate increase in atrial effective refractory period (AERP) and decrease in AERP dispersion, which suggests the potential reversibility of the electrophysiological features of chronic atrial dilatation. Later on, Coronel et al.^17^ determined AERP and activation times in 9 patient before and after PBMV and concluded that MS is associated with LA conduction delay, increased LA dispersion of conduction, and conduction asymmetry. These electrophysiologic changes were immediately reversed by PBMV. There are only two studies which have explored the relation between MS and P-wave indices. First, Turhan et al.^6^ studied 29 patients who were undergoing PBMV and found that P_max_ and PWD were significantly increased in patients with severe MS than in healthy control subjects and both decrease progressively after PMBV. Later, Guntekin et al.^7^ followed 30 patients with mild to moderate MS and observed that P_max_ and PWD increase progressively in accordance with increasing severity of MS.

Rheumatic MS is an important health issue in the developing countries.^5^ Increased LA pressure due to MS and the inflammation secondary to rheumatic carditis may produce atrial stretch and dilatation, fibrotic changes within the wall of the atrium and disorganization of the atrial muscle bundles.^18^ These structural changes may lead to unequal conduction velocities and heterogeneous refractory periods through the atrial myocardium^5,18^ reflecting on ECG as increased P_max_ and PWD which lead to AF.^1,2^ In fact, AF is the most common sustained arrhythmia encountered in these patients which is poorly tolerated due to both loss of atrial contraction and associated rapid ventricular rate, and increases morbidity and mortality.^5^ Timely and most importantly “effective” PBMV may help in preventing the progress of the above electrical and structural atrial remodeling and even reverse them, leading to a delay or even prevention in the initiation of AF.

Our findings complement previous studies on the immediate and short term electrophysiologic effects of relieving mitral valve obstruction reflected by simple ECG indices. The new finding of this study is that P_max_ and PWD are reduced only if there is effective relief of mitral valve obstruction leading to decreased LA pressure (Tables 2, 3). In fact PWD was increased at follow-up of patients with unsuccessful PBMV although it did not reach statistical significance. This happened despite similar age, valve scores, and reduction in sPAP after both successful and unsuccessful procedures (Table 1). The reason was either an insufficient increment in MVA and/or an increase in the degree of MR after PBMV leading to continued left atrial hypertension. The trend for less reduction in mMVG after unsuccessful PBMV (Table 1) may support this idea.

Another finding in our study was that in patients with a successful PBMV the decreases in P_max _and PWD continued progressively and were not significantly correlated with improvements of echocardiographic variables (MVA and mMVG) at the end of the first month follow-up. Previous studies^6,15^ have also shown this discrepancy between P-wave indices and echocardiographic variables. Some investigators have reported increased sympathetic activity in patients with hemodynamically significant MS^19,20^ which could lead to a significant increase in PWD.^21^**** Cheema et al.^22^ verified significant prolongation of P_max_ in healthy subjects during epinephrine infusion. Ashino et al.^19^ demonstrated that PBMV results in early and long-lasting normalization of sympathetic nerve activity, possibly because of an improvement in arterial baroreflex sensitivity associated with increased cardiac index. We agree with Turhan et al.^6^ that this might be the main reason for the immediate reduction in P_max _and PWD after successful PBMV. The continued decrease in P_max _and PWD during follow-up may be due to remodeling and regression of the LA wall due to decreased intracavitary pressure leading to more homogeneous and continuous propagation of sinus impulses.

A striking difference in our study compared to most others is a relatively low success rate of 73.1% after PBMV. The best results from PBMV are expected with favorable valve morphology, no or mild MR, and no evidence of left atrial thrombus. The Wilkins score gives a rough guide to the suitability of the mitral valve’s morphology for PBMV.^8^ A score of 8 or lower is usually associated with an excellent immediate and long-term result, whereas scores exceeding 8 are associated with less impressive results. In the present study the mean valvular score in all patients was 8.5±1.2 (range: 5-12). This high score can certainly be related to the relatively low success of our procedures. Our hospital is a tertiary referral center with a high volume of PBMV performed over the past 15 years. Recently, we reported our results in 1745 patients who underwent PBMV between 1999 and 2009.^10^ Pathan et al.^23^ included older patients at a mean age of 58 years with more deformed valves, 50% having a Wilkins echo score >8, and 77% having valve calcification, with an immediate success rate of 75%. Turgeman et al.^24^ included patients at a mean age of 45 years with echo findings similar to Pathan’s study^23^ and achieved an immediate success rate of 82%. When we reanalyzed our patients according to a score≤ 8 (16 patients) or >8 (34 patients), PBMV was successful in 78.9% of those with a score≤ 8 with respect to 67.6% in those with a score> 8. These results are more in accordance of previous studies.

## Limitations

There were several limitations in this study. First, the sample size was relatively small, which highlights the need for larger studies. Second, the measurements performed manually on paper-printed ECGs using magnifying lens instead of computer assisted P-wave calculations may restrict the accuracy and reproducibility of the measurements. Although several studies have demonstrated a low error of the measurement of PWD on paper-printed ECGs,^12,21^ others have questioned the accuracy and reproducibility of this method.^25,26^ Third, large-scale and long-term studies may be necessary to assess the clinical impact of decrease in P_max_ and PWD on prevention of AF.

## Conclusion

Only successful PBMV was associated with a decrease in P_max _and PWD. These simple ECG indices may predict the success of the procedure immediately after PBMV. Patients without any decrease in Pmax and PWD after PBMV may potentially be at greater future risk and deserve closer follow-up.

## Acknowledgments

This article was written based on a dataset of specialty in cardiology thesis, registered in Tabriz University of Medical Sciences.

## Ethical issues

Informed consent was obtained from each patient and the study protocol conformed to the ethical guidelines of the 1975 Declaration of Helsinki as reflected in a priori approval by the institution’s human research committee.

## Competing interests

The authors declare that they have no conflict of interest.
